# Combined talimogene laherparepvec and binimetinib in *NRAS*-mutated melanoma induces antitumor immunity

**DOI:** 10.1172/JCI211137

**Published:** 2026-09-29

**Authors:** Tony Yao, Rita E. Chen, Melissa Yamada, J. Russell Moore, Marissa Jimenez, Tammy Huang, Lynn Cornelius, George Ansstas, Naresha Saligrama, David Y. Chen

**Affiliations:** 1 Department of Medicine,; 2 Division of Dermatology,; 3 Department of Neurology,; 4 Division of Oncology, and; 5 Department of Pathology and Immunology, Washington University, St. Louis, Missouri, USA.

**Keywords:** Dermatology, Immunology, Oncology, Cancer immunotherapy, Melanoma, Skin cancer

## Abstract

Combined oncolytic viral therapy with MEK inhibition is associated with unexpectedly strong responses in checkpoint-failed NRAS-mutated melanoma

**To the Editor:** Combined immune checkpoint blockade (ICB) produces durable survival benefit in over half of metastatic melanoma patients, but many will require subsequent lines of therapy (1). Talimogene laherparepvec (T-VEC) is an oncolytic immunotherapy derived from herpes simplex virus type 1 and is approved as a local therapy for unresectable melanoma. Real-world data suggest that it remains effective for treatment of localized disease after ICB failure (2). While T-VEC can induce immune responses, it does not consistently produce clinical responses in noninjected lesions and is generally used as locoregional therapy. We observed, however, that T-VEC combined with binimetinib induced exceptional clinical responses in a series of patients with *NRAS*-mutated melanoma after ICB failure, including patients with noninjectable disease.

Our index patient was a woman with stage IIIC melanoma of the right ankle treated with resection and 1 year of adjuvant nivolumab, followed 7 months later by cutaneous recurrence. She was treated with ipilimumab and nivolumab but developed grade 4 interstitial nephritis, requiring discontinuation after 2 cycles. At referral for progressive disease (Figure 1A, patient 1), we initiated T-VEC and obtained tumor molecular profiling, which identified an *NRAS*^Q61R^ mutation. Due to clinical progression, we added binimetinib before the fourth T-VEC cycle, resulting in rapid tumor regression. T-VEC was discontinued after 10 cycles, and binimetinib was held 2 months later due to rhabdomyolysis; she remains on observation with complete response (CR).

Similar responses emerged among subsequent patients with checkpoint-failed, *NRAS*-mutated melanoma. In our observational cohort, 6 of 7 patients (85.7%) receiving T-VEC and binimetinib (“combination therapy”) achieved a CR compared with 2 of 7 (28.6%) treated with T-VEC alone or with ICB (Figure 1B), consistent with prior observations in unselected patients (2). Notably, CR in the combination therapy group occurred despite having rapidly progressive disease (denoted as red events in Figure 1B and Supplemental Case Descriptions; supplemental material available online with this article; https://doi.org/10.1172/JCI211137DS1) and a higher frequency of distant, noninjected lesions (71.4% versus 14.3%) and brain metastases (42.9% versus 0%; Supplemental Table 1). These outcomes are not expected from MEK inhibition alone, which in *NRAS*-mutated melanoma is associated with a 15% overall response rate, rare CRs, and a median progression-free survival of 2.8 months (3).

We conducted single-cell profiling of paired pretreatment and T-VEC–injected tumor biopsies from patients 2, 3, and 4. Among CD8^+^ T cells, expansion of cytotoxic effectors on treatment displaced the existing terminally exhausted population (Figure 1, C and D). Paired TCR sequencing demonstrated this phenotypic shift across pretreatment CD8^+^ T cell clonotypes from all patients (Figure 1E and Supplemental Figure 1, A and B), reflecting renewal of preexisting clones rather than clonal replacement as reported after ICB (4). Cytotoxic, exhausted, and progenitor CD8^+^ T cell clusters exhibited a CD39^+^CD103^+^ tumor-reactive phenotype (Figure 1F). Pretreatment exhausted CD8^+^ T cells had increased LAG-3 expression and reduced expression of transcriptional programs associated with effective TCR signaling (Figure 1G and Supplemental Figure 1C). In patient 2, we identified CD8^+^ clones with highly similar TCR sequences, consistent with convergent recognition of a shared antigen (Supplemental Figure 1E). We expressed 2 TCRs from this group in primary CD8^+^ T cells and cocultured these with an autologous melanoma cell line (Supplemental Figure 1F). Both preexisting (TCR-42) and treatment-emergent (TCR-157) clones from this group demonstrated autologous tumor reactivity (Figure 1H).

On therapy, intratumoral CD4^+^ T cells were enriched for a CXCR6^+^ Th1 subset distinct from the follicular helper (Tfh) phenotype associated with tumor-reactive CD4^+^ T cells (Supplemental Figure 2, A–D). Preexisting CD4^+^ clones mostly retained their prior Tfh or Treg phenotypes on treatment, whereas Th1 cells predominantly comprised newly detected clonotypes (Supplemental Figure 2E). A small fraction of stem-like cells could be found in both Th1- and Tfh-biased clones (Supplemental Figure 2, F and G). We observed no consistent changes in macrophage or dendritic cell subsets. However, B cells and IgG1^+^ antibody-secreting cells expanded, and endothelial cells upregulated interferon-inducible genes (Supplemental Figure 3).

Our findings suggest that binimetinib may amplify and broaden T-VEC–associated antitumor immunity in *NRAS*-mutated melanoma. The renewal of existing CD8^+^ T cell clonotypes and emergence of new CD4^+^ Th1 clones suggest that T cell dynamics on combination therapy may be distinct from those described following ICB (4, 5). This potentially indicates an orthogonal mechanism of immune activation, which is consistent with many T-VEC–based therapy responses occurring in the setting of prior ICB failure (2). Whether CD4^+^ Th1 cells recognize tumor antigens, viral antigens, or both, and whether they support tumor-reactive CD8^+^ T cells, remain important open questions.

Patient 7, the only nonresponder to combination therapy, had progressed on binimetinib prior to starting T-VEC, suggesting that tumor sensitivity to MEK inhibition may contribute to response. Thus, tumor-intrinsic sensitivity, immune remodeling, and augmented oncolytic infectivity and immune activation by MEK inhibition may all contribute to the depth and durability of antitumor responses (6).

This study is limited by its retrospective, single-center design, and immunologic analyses were limited to T-VEC–injected tumors. Prospective studies are needed to test the efficacy of combined T-VEC and binimetinib for *NRAS*-mutated melanoma and whether responses extend to melanoma, or other tumors, with other MAPK pathway mutations. Correlative studies in prospective trials should examine injected tumors, noninjected tumors, and peripheral blood to determine whether binimetinib enhances systemic immunity by augmenting tumor-reactive T cell priming, function, or clonal diversity; remodels vascular, stromal, or myeloid compartments of the tumor microenvironment; or induces tumor-intrinsic changes that enhance immune recognition.

For detailed methods, information regarding sex as a biological variable, statistics, study approval, data availability, and author contributions, see the supplemental materials.

## Manuscript information

### Conflict of interest.

DYC has served as a consultant for Replimune.

### Funding.

This study was supported by the following:

- NIH grant (K08 CA237727 to DYC).
- Two Sisters Foundation grant (MJ).
- Alvin J. Siteman Cancer Center Catalyst Award (NS).
- Washington University School of Medicine Departments of Neurology (NS) and Medicine (DYC) start-up funds.
- Canadian Institutes of Health Research Doctoral Foreign Study Award (TY).

This work is supported in part by NIH funding and is subject to the NIH Public Access Policy. Through acceptance of this federal funding, the NIH has been given a right to make the work publicly available in PubMed Central.

## Supplementary Material

Supplemental data

Supporting data values

## Figures and Tables

**Figure 1:**
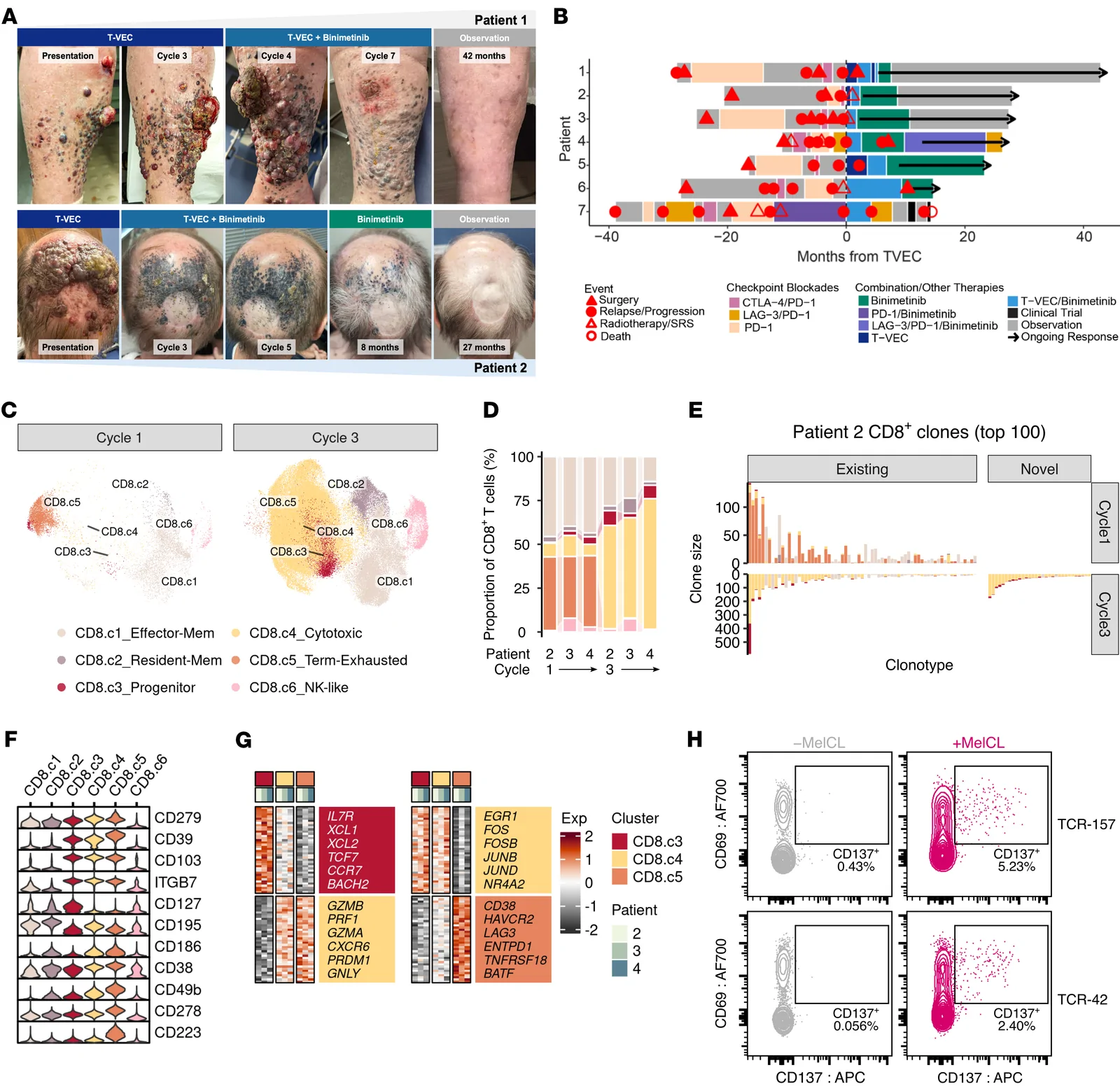
Combined T-VEC and binimetinib therapy revives antitumor CD8^+^ T cell immunity. (**A**) Clinical response with combination therapy in patients 1 and 2. (**B**) Clinical summary of all cases. (**C**) Uniform manifold approximation and projections (UMAPs) of pre- and on-treatment intratumor CD8^+^ T cells from patients 2–4. Mem/Term, memory/terminal. (**D**) Pre- and on-treatment phenotypic distribution of CD8^+^ T cells. (**E**) Phenotypic distribution among expanded CD8^+^ T cell clones from patient 2. (**F**) Surface protein expression across CD8^+^ T cell subsets. Violin plots show the full distribution of the data, with the width representing the density of values. (**G**) Differentially expressed genes between progenitor, cytotoxic, and terminally exhausted CD8^+^ T cells. (**H**) TCR-engineered CD8^+^ T cell activation with or without exposure to autologous melanoma cells.

## Version 1 09/29/2026

In-Press Preview

## Version 2 11/16/2026

Electronic publication
