# Supplementary material for: Combined talimogene laherparepvec and binimetinib in NRAS-mutated melanoma induces antitumor immunity
Source: J Clin Invest. 2026 Sep 29;136(22):e211137. doi: 10.1172/JCI211137 (PMC13767615; doi:10.1172/JCI211137)
Supplement: Supplementary file 1 [file jci-136-211137-s251.pdf]

SUPPLEMENTAL INFORMATION

Combined talimogene laherparepvec and binimetinib in *NRAS*-mutated melanoma induces anti-tumor immunity

Tony Yao[1,2]*, Rita E Chen[1,3]*, Melissa Yamada[1], J Russell Moore[1], Marissa Jimenez[1,3], Tammy Huang[4], Lynn Cornelius[1,3,9], George Ansstas[1,5,7,9#], Naresha Saligrama[4,6,7,8,9#], David Y Chen[1,3,9#]

Affiliations:

1. Department of Medicine, Washington University School of Medicine, St. Louis, MO
2. Medical Scientist Training Program, Washington University School of Medicine, St. Louis, MO
3. Division of Dermatology, Washington University School of Medicine, St. Louis, MO
4. Department of Neurology, Washington University School of Medicine, St. Louis, MO
5. Division of Oncology, Washington University School of Medicine, St. Louis, MO
6. Pathology and Immunology, Washington University School of Medicine, St. Louis, MO
7. Bursky Center for Human Immunology and Immunotherapy, Washington University School of Medicine, St. Louis, MO
8. Hope Center for Neurological Disorders, Washington University School of Medicine, St. Louis, MO
9. Siteman Cancer Center, St. Louis, MO

***Equal contribution**
#Co-corresponding

Contents:

1. Clinical case descriptions of patients 1-7
2. Methods
3. Table S1
4. Figures S1-S3
5. Supplemental References

Acknowledgements: We are deeply grateful to the patients and their families for their participation and making this work possible.

Author contributions: T.Y. and R.E.C. performed sample processing, experiments, and data analyses. M.Y., J.R.M., and M.J. performed clinical data collection. T.H. performed experiments. G.A., N.S., and D.Y.C. conceived the project and supervised the research. T.Y. and R.E.C. wrote the initial manuscript draft. T.Y., R.E.C., L.C., N.S., and D.Y.C. edited the manuscript.

CASE DESCRIPTIONS

Patient 1: A 68-year-old woman presented with concurrent superficial spreading melanoma, Breslow thickness 1.1mm, on the right ankle and superficial spreading melanoma, Breslow thickness 0.2mm, on the back. Three weeks later, both lesions were excised with sentinel lymph node biopsy revealing 4/8 examined nodes positive for melanoma, and staging imaging, including brain MRI, were negative for metastatic disease. She underwent adjuvant nivolumab therapy for one year. Seven months later, she developed a right ankle in-transit melanoma, negative for $BRAF^{V600E}$ mutation, which was excised 12 weeks later. Three weeks following excision, she initiated ipilimumab plus nivolumab checkpoint blockade therapy, which was complicated by grade 4 acute interstitial nephritis causing treatment cessation after cycle 2. Follow-up PET-CT demonstrated metastatic right external iliac chain disease, and she was offered, but declined, palliative chemotherapy. She then presented to our multidisciplinary clinic where we performed a biopsy of a right shin lesion that we sent for next generation sequencing (**Figure 1A, top**). She initiated T-VEC therapy at this visit, and after four cycles of T-VEC, her leg lesions continued to expand and become increasingly symptomatic. Dermatologic surgery was consulted for palliative debulking surgery for the largest, most symptomatic nodule. The day prior to debulk, she added binimetinib in combination with T-VEC after sequencing results were positive for a $NRAS^{Q61R}$ mutation (BRAF WT, MAP2K1/MAP2K2 WT). Surprisingly, much of the debulked mass was necrotic with <10% viable tumor. After 2 months of combined T-VEC and binimetinib, she developed hematuria, and binimetinib was held for 3 weeks before re-initiating at a reduced dose. She continued with T-VEC for 10 total cycles (loading dose 1ml of 10^6 PFU/ml to 3 cutaneous lesions, followed by 4ml of 10^8 PFU/ml to 25 to 40 cutaneous lesions for all subsequent doses), after which she developed edema and erosions at the injection sites, so T-VEC was discontinued. Less than two weeks after that, she developed rhabdomyolysis and binimetinib was also discontinued. Since then, she has been on clinical observation and continues to have no evidence of disease 42 months after starting T-VEC.

Patient 2: A 70-year-old man was found by his dermatologist to have a 1.9mm Breslow thickness, non-ulcerated melanoma on the right upper back for which he underwent wide local excision with sentinel node biopsy with one of three nodes positive for melanoma, negative for BRAF V600E mutation. Staging, including brain imaging, did not reveal metastatic disease, so he elected for clinical surveillance with imaging and circulating tumor DNA blood screening. Fourteen months later, he was found to have cutaneous metastatic melanoma on the scalp, which was excised, followed by initiation of adjuvant nivolumab. At this time his tumor was sent for sequencing, revealing an $NRAS^{Q61R}$ mutation (BRAF WT, MAP2K1/MAP2K2 WT). He was found to have significant cutaneous progression of the scalp after two cycles of nivolumab (**Figure 1A, bottom**). CT head and neck revealed new nodules on the right posterior scalp and neck representing metastatic disease, and brain MRI revealed new right posterior temporal lobe and right superior occipital lobe lesions suspicious for metastatic disease, which prompted initiation of combined ipilimumab and nivolumab checkpoint blockade. Given continued cutaneous progression, ipilimumab and nivolumab were discontinued, and he was referred to our multidisciplinary clinic where we proceeded with T-VEC and binimetinib. One month after T-VEC initiation, he received gamma knife stereotactic radiosurgery (SRS) to his brain metastases. He experienced rapid clinical response, and after 5 cycles of T-VEC (loading dose 1ml of 10^6 PFU/ml to 10 cutaneous lesions followed by 2 ml of 10^8 PFU/ml to 1-10 cutaneous lesions for subsequent doses) we performed biopsies revealing pathological complete response with tumoral melanosis, and T-VEC was discontinued. During this time, he was also noted to have an acneiform rash, a well-described cutaneous side effect of MEK inhibitors. He also developed dropped head syndrome, which is an uncommon side effect of MEK inhibitor; binimetinib was discontinued 6 months later, resulting in complete resolution of his cervical

myopathy. He remains without evidence of disease on observation 27 months after starting T-VEC and binimetinib.

Patient 3: A 34-year-old man presented with a lesion on the right forearm with a 1.1mm Breslow thickness Spitzoid melanoma for which he underwent wide local excision and sentinel lymph node biopsy two months later with 1/1 examined lymph nodes positive for melanoma. He then underwent one year of adjuvant nivolumab therapy. Fourteen months later, he developed a recurrent melanoma in his right forearm scar for which he underwent wide local excision with sentinel lymph node biopsy where 0/4 examined nodes were positive. One month later, he developed an occipital metastatic melanoma lesion. PET-CT revealed a new, hypermetabolic right level 5 cervical lymph node suspicious for metastatic disease while brain MRI was negative for intracranial metastases. He underwent neoadjuvant ipilimumab plus nivolumab for 2 cycles followed by scalp lesion resection and right neck dissection, finding 2/69 nodes positive for melanoma. One month later, PET-CT revealed new FDG-avid nodules on his right forearm that were biopsied, revealing recurrent melanoma. Sequencing demonstrated $\textit{NRAS}^{Q61K}$ mutated (BRAF WT, MAP2K1/MAP2K2 WT) melanoma, prompting binimetinib initiation followed by T-VEC the following week. Decreased nodularity in the right forearm was noted after four cycles of T-VEC (loading dose 1ml of 10^6 PFU/ml to 2 cutaneous lesions followed by 0.3 - 0.5 ml of 10^8 PFU/ml to 1-2 cutaneous lesions for subsequent doses), and biopsies revealed complete pathologic response. After 11 months of binimetinib, his left ventricular ejection fraction (LVEF) was noted to be 43%, and binimetinib was held. His LVEF improved to 51% after 1 month of binimetinib discontinuation. He is under observation now and has continued to show no evidence of recurrence for 27 months after starting T-VEC and binimetinib.

Patient 4: A 63-year-old woman presented with a left lower extremity mass that had been growing over 6 months. Punch biopsy revealed an ulcerated, 20mm Breslow thickness nodular melanoma. Staging brain MRI revealed a hemorrhagic right parieto-occipital brain mass, and both primary melanoma and brain lesions were surgically resected. After pathologic confirmation of brain metastatic melanoma, she underwent SRS to an intact brain lesion and radiotherapy to the resection bed. She then completed four cycles of ipilimumab and nivolumab, but restaging PET-CT two weeks later revealed an early recurrence at the left lower extremity, which was resected three weeks later while on maintenance nivolumab. MRI two weeks following identified brain metastases for which she received additional SRS while on nivolumab. At this time, she switched to relatlimab in combination with nivolumab, but three months later, biopsy of the left lower leg demonstrated recurrent melanoma which was sent for sequencing, revealing an $\textit{NRAS}^{Q61K}$ mutation (BRAF WT, MAP2K1/MAP2K2 WT). She then presented to our multidisciplinary clinic and started binimetinib in combination with T-VEC. After six cycles of T-VEC (loading dose 1ml of 10^6 PFU/ml to 1 cutaneous lesion followed by 2 ml of 10^8 PFU/ml to 1 cutaneous lesion for subsequent doses), she achieved complete cutaneous response, and T-VEC was stopped. Three months later, she developed a left frontal lobe recurrence while on binimetinib for which she underwent surgical excision and SRS. Two months later, she restarted nivolumab and relatlimab in addition to binimetinib. Binimetinib was discontinued 13 months later, and she continues to have no evidence of progression at 26 months since T-VEC initiation.

Patient 5: A 55-year-old woman presented with a right second toe 1.8mm Breslow thickness nevoid acral melanoma without ulceration. She then underwent ray amputation, but a sentinel lymph node biopsy was not performed due to lack of tracer activity. CT imaging was negative for distant disease. She remained on surveillance for six years until she developed an in-transit melanoma on her right foot which was excised, and PET-CT was negative for residual or distant disease. However, a new nodule had developed over the excision scar a month later, prompting her to complete 7 cycles of single agent nivolumab over 8 months but stopped due to clinical evidence of recurrence along her right shin. Three months later, she began treatment with

ipilimumab plus nivolumab but stopped after two cycles when she developed grade 4 immune-mediated colitis, which required treatment with infliximab and a high dose prednisone taper. Three months after discontinuing immunotherapy, PET-CT demonstrated the presence of at least five FDG-avid lesions on the patient's right shin consistent with progressive disease. A month afterwards, she initiated T-VEC therapy and completed 5 cycles (loading dose 1ml of 10^6 PFU/ml to 5 cutaneous lesions followed by 0.3 – 1 ml 10^8 PFU/ml to 3-7 cutaneous lesions for subsequent doses) in 3 months with some systemic side effects, including fever and malaise. Imaging demonstrated disease progression with development of a new hypermetabolic subcutaneous nodule on her right shin and increased size and metabolic activity of injected lesions. She then started binimetinib for $NRAS^{Q61R}$ mutated (BRAF WT, MAP2K1/MAP2K2 WT) melanoma and completed six additional, concurrent cycles of T-VEC over the next 4 months, achieving a complete response. During this time, binimetinib was held for less than a month due to elevations in her hepatic enzyme labs. She also developed an acneiform eruption in response to binimetinib, which was treated with doxycycline. Restaging PET-CT after seven total months of T-VEC injections revealed minimal residual FDG avidity along the right anterior tibia. Biopsy demonstrated changes suggestive of treatment effect. She continues to have no new or progressive disease while still on binimetinib 23 months after starting T-VEC.

Patient 6: A 44-year-old woman presented with a 1.1mm Breslow thickness, ulcerated melanoma of the right forearm that had been increasing in size for over a year. One month later, she underwent wide local excision with sentinel lymph node biopsy where 0/3 examined lymph nodes were positive for melanoma, and the melanoma was found to be positive for a NRAS^{Q61K} mutation (BRAF WT, MAP2K1/MAP2K2 not tested) by next generation sequencing. Subsequent imaging, including a brain MRI, revealed no evidence of metastatic disease. A year later, the patient presented with pain and purulent drainage from a lesion in her right axilla. She underwent a right axillary lymph node biopsy, which revealed metastatic melanoma. Two weeks later, a brain MRI identified a left occipital lesion, and PET-CT showed a right axillary necrotic lymph node but no other sites of uptake. She then started ipilimumab and nivolumab therapy, which was held after three cycles due to hepatopathy requiring prolonged prednisone therapy. PET-CT two months later showed decreased metabolic activity in the previously identified right axillary lymph node but identified a new site of uptake in the right femur. She then started single agent nivolumab and after six cycles, PET-CT demonstrated disease progression and brain MRI revealed a new left cerebellar lesion. Two months later, she underwent SRS of her brain lesions and began treatment with T-VEC injections combined with binimetinib. After completing 16 cycles of T-VEC (loading dose 1ml of 10^6 PFU/ml to 1 axillary lymph node followed by 0.5 – 1 ml 10^8 PFU/ml to 1 axillary lymph node for subsequent doses), imaging studies suggested decreased right axillary uptake and no evidence of distant disease. She then underwent surgical resection of her right axillary lymphadenopathy, and no viable tumor was identified; she achieved a complete response which continues to persist 14 months after starting T-VEC therapy.

Patient 7: A 71-year-old woman with a distant history of cutaneous melanoma (> 20 years prior) on the right arm and right lateral lower leg presented with a right inguinal metastatic melanoma diagnosed by core biopsy. A right, superficial inguinal node dissection revealed 2/18 examined lymph nodes positive for metastatic disease, and PET-CT three months later showed no evidence of recurrent or residual disease. However, 6 months afterwards, repeat PET-CT demonstrated a metastatic lesion in the subcutaneous tissue of the anterior right thigh, which was excised. PET-CT around 17 months later revealed a pulmonary nodule and left adrenal gland lesion, both highly suspicious for metastatic disease. While the patient initially elected for surveillance, she opted to begin treatment 5 months later with single agent nivolumab. After 3 months, CT imaging demonstrated disease progression, so she was transitioned to nivolumab and relatlimab. After 5 months, she switched to ipilimumab and nivolumab due to disease

progression, completed 4 cycles, and underwent a left adrenalectomy, followed by SBRT to pulmonary nodules. At this time molecular profiling revealed *NRAS*^{Q61R} mutation (BRAF WT, MAP2K1/MAP2K2 WT). While on maintenance nivolumab, around 7 months later, imaging identified a new nodule within the left adrenalectomy bed suspicious for recurrent disease, so she received SBRT to this adrenal nodule and started on binimetinib in combination with nivolumab. After a year, imaging demonstrated right inguinal nodal progression, so T-VEC was initiated for the right inguinal metastatic disease combined with binimetinib and nivolumab. She completed 9 cycles of T-VEC (0.5 ml loading dose of 10^6 PFU/ml to single right inguinal lymph node, followed by 0.5ml -1ml of 10^8 PFU/ml to single right inguinal lymph node for subsequent doses) over the course of 4 months and follow up CT imaging demonstrated disease progression in the chest in addition to new hepatic and gallbladder lesions suspicious for metastases. She discontinued T-VEC and completed 4 cycles of nivolumab and relatlimab, but experienced disease progression. She then participated in a clinical trial in which she received CD137 agonist CTX-471 at 0.1 mg/kg every 2 weeks and pembrolizumab every 6 weeks. She discontinued the trial about three months later when CT imaging confirmed disease progression. She then began treatment on a clinical trial with BTLA antagonist TAB004 and toripalimab, completing two cycles of treatment before presenting with a left cerebellar hemorrhagic metastasis, resulting in her death.

In summary, this series of patients with *NRAS*-mutated melanoma has experienced unexpectedly high rates of favorable response to combination T-VEC and binimetinib. In cases where T-VEC was initiated with clinical or radiographic evidence of progression (Patients 1 and 5), addition of binimetinib subsequently resulted in durable CRs. One patient (Patient 4) had a brain recurrence after complete response of the injected cutaneous lesion; however, after craniotomy and resection, has had no evidence of recurrence, which contrasts with the rapid progression through multiple therapies prior to T-VEC and binimetinib. While limited recurrences have been observed in responders (Patients 1-6), the overall decreased frequency of recurrence events after combination therapy suggests that the course of disease was altered in these patients. The specific combination with binimetinib may also be consequential, distinguishing this cohort from other experiences with MEK inhibitors combined with T-VEC (1).

METHODS

Sex as a biological variable. This cohort included both female and male patients, reflecting consecutive patients treated on a standard-of-care basis at our comprehensive cancer center. Sex was considered as a biological variable, but small sample size precluded formal assessment of sex-specific differences in response, toxicity, or immune correlates.

Case selection and response criteria. Eligible patients for this retrospective cohort study were aged ≥18 years who had confirmed *NRAS* mutations by next generation sequencing, had received three or more doses of T-VEC therapy, and completed T-VEC-based therapy at Washington University School of Medicine in St. Louis and Siteman Cancer Center. Eligible patients started T-VEC based therapy between January 1, 2018 and January 1, 2025 with a data cutoff date of December 15, 2025 to ensure adequate time for durable clinical response determination. Of 92 patients treated in this time frame, a total of 14 patients met these eligibility criteria. No patients were excluded. We used investigator-assessed objective response (OR) for response determination as previously described (main text ref 2). Briefly, progressive disease (PD) was determined by increasing number or growth of lesions necessitating a switch in therapy, while complete response (CR) was defined by clinical or radiographic resolution of targetable lesions. All CRs in this study were confirmed by target lesion histopathology.

Correlative specimen collection. Tumor punch biopsies and peripheral blood were collected from patient participants who provided informed consent prior to T-VEC initiation and prior to cycle 3 of T-VEC.

Tumor processing. Fresh tumor specimens were minced and digested in RPMI supplemented with 20% fetal bovine serum (FBS), collagenase type IV (Worthington CLS-4), and Benzonase (Sigma 70664) for 2 hours at 37°C, then manually dissociated through a 70-μm cell strainer to obtain single cell suspensions. Cells were cryopreserved in 10% DMSO.

Single cell sequencing. Cryopreserved pre-cycle 1 and pre-cycle 3 tumor biopsy cell suspensions from three patients (Patients 2-4) were thawed, stained, and sorted for cellular indexing of transcriptomes and epitopes (CITE-seq) with joint single cell T and B cell receptor sequencing (scTCR/BCR-seq). Thawed cells were stained with Zombie Green viability dye (BioLegend 423112), blocked with human TruStain FcX solution (BioLegend 422302), and labelled with oligo-conjugated TotalSeq-C (BioLegend), respective hashing (BioLegend 394661, 394663, 394665, 394667, 394669, 394671), and BV421 anti-human CD45 (clone HI30, Biolegend 304032) and PE anti-human CD31 (clone WM59, BioLegend 303106) sorting antibodies in PBS with 0.5% bovine serum albumin (BSA). Live singlet $CD45^+$ immune and $CD45^-$ $CD31^+$ endothelial cells were sorted using a BD FACSAria II, pooled, and resuspended in PBS with 0.04% BSA. Single cell partitioning, cDNA amplification, and generation of gene expression, V(D)J, and feature barcode libraries were performed according to the Chromium Next GEM Single Cell 5' HT v2 protocol (10x Genomics CG000424). Libraries were sequenced on Illumina Novaseq X Plus systems by the Washington University Genome Technology Access Center.

Pre-processing of CITE-seq data. FASTQ files from gene expression and feature barcode libraries were respectively aligned to the GRCh38.p13 genome assembly (GENCODE v32) and a supplied TotalSeq-C feature reference, counted, and demultiplexed using Cell Ranger v7.2.0 multi (2) to generate count matrices for each GEM well. Feature-barcode matrices were imported and merged into a Seurat v5.0.0 object (3). Cell barcodes were filtered based on the number of detected gene expression features (300 < *nFeature_RNA* < 8,000), non-hashtag feature barcode UMIs (500 < *nCount_ADT* < 30,000), hashtag UMIs (200 < *nCount_HTO* < 20,000), percent of mitochondrial gene counts (*percent.mt* < 10%), and percent of ribosomal gene counts (*percent.rb* > 3%). Cells were assigned to their sample of origin (patient ×

timepoint) and hashtag-heterotypic doublets were excluded using the MULTIseqDemux function (quantile = 0.3) (4) on HTO counts. Feature barcode UMI counts were denoised and normalized using the DSBNormalize function (dsb v1.0.3) (5) with quantile clipping at 0.005 and 0.9999.

Dimensionality reduction, clustering, and visualization of CITE-seq data. Raw gene expression UMI counts were log-normalized and scaled. Principal component analysis (PCA) reductions were separately performed on the top 3,000 variable RNA features and DSB-normalized surface protein data. RNA assay data from different subjects were integrated using the Seurat reciprocal PCA method. A weighted-nearest neighbor graph was computed with the first 30 RNA PCs and first 20 DSB PCs, which was used as input for smart local moving clustering and UMAP visualization. This clustering procedure was applied first to categorize cells into broad lineages ($CD8^+$ T, $CD4^+$ T, NK, B/plasma, myeloid, endothelial cells), then iteratively on each subset to assign granular cell type labels. Differentially expressed genes between cell groupings were identified using Seurat implementation of Wilcoxon rank-sum tests and visualized with ComplexHeatmap v2.20.0 (6) as pseudobulk log-normalized data scaled by row. All other visualizations were generated with ggplot2 v3.5.1 or Seurat functions.

Processing and visualization of scTCR-seq data. FASTQ files from V(D)J libraries were assembled and annotated based on the prebuilt GRCh38_alts_ensembl-5.0.0 reference using Cell Ranger v7.2.0 multi to generate clonotype tables for each GEM well. High-confidence contig annotations (filtered_contig_annotations.csv) were appended as metadata to T cells in the CITE-seq dataset. For each patient, clones were defined as cells sharing TCRα/β chains with fully matched V(D)J gene segments and CDR3 nucleotide sequences, permitting α chain dropout only in cases where the β chain sequence is uniquely identifying. Clonotypes were categorized as existing if recovered from both pre-cycle 1 and cycle 3 timepoints and as novel if found only at cycle 3. Phenotypic distributions of expanded clones, ranked by cumulative frequency across timepoints, were visualized using ggplot2 v3.5.1.

Generation of primary melanoma cell line. In parallel to sorting immune and endothelial for single cell sequencing, live $CD45^-$ $CD31^-$ cells were sorted from pre-cycle 1 tumor biopsies to enrich melanoma cells for culture. Sorted cells were expanded and passaged in Opti-MEM (Gibco 51985034) supplemented with 5% FBS, 1 mM sodium pyruvate, 100 U/mL penicillin-streptomycin, 5 µg/mL insulin (Santa Cruz Biotechnology, sc-360248), and 5 ng/mL recombinant human EGF (Gibco, PHG0311). A primary melanoma cell line was derived from patient 2 and aliquots of early-passage cells were cryopreserved. Whole-exome sequencing of genomic DNA from this cell line confirmed presence of the $\textit{NRAS}^{Q61R}$ driver mutation.

TCR engineering in primary T cells. Paired TCRα/β sequences were selected from expanded $CD8^+$ T cell clones in Patient 2 and cloned as polycistronic TCRβ-P2A-TCRα inserts into the pLVX-EF1α-IRES-Puro lentiviral vector (Takara, 631988) by GenScript. Murine *Trac* and *Trbc1* sequences with stabilizing cysteine substitutions (7) were used for TCR construct constant regions. For viral packaging, Lenti-X 293T cells (Takara, 632180) were transfected using VSV-G Single Shots (Takara, 631282), then 48 h supernatants were collected, concentrated with Lenti-X Concentrator (Takara, 631231) per manufacturer's protocols, and used without freezing.

Human $CD8^+$ T cells were purified from PBMCs by magnetic negative selection (StemCell, 17953) and activated for 48 h in complete RPMI (cRPMI) containing 10% fetal bovine serum (FBS), 1 mM sodium pyruvate, 1× non-essential amino acids, and 100 U/mL penicillin-streptomycin supplemented with 25 ng/mL IL-7 (Miltenyi, 130-095-367), 50 ng/mL IL-15 (Miltenyi, 130-095-760), and TransAct anti-CD3/CD28 reagent (Miltenyi, 130-128-758). Immediately prior to transduction, endogenous TCR chains were disrupted in stimulated $CD8^+$ T cells by CRISPR/Cas9 nucleofection (8). Ribonucleoprotein complexes (RNPs) were formed by mixing 180 pmol each of single guide RNA targeting human *TRAC* (IDT,

TCAGGGTTCTGGATATCTGT) and *TRBC1/2* (IDT, CGTAGAACTGGACTTGACAG) with 60 pmol TrueCut Cas9 v2 (Invitrogen, A36499) and incubating for 10 min at room temperature. Up to 5 million stimulated CD8$^+$ cells were resuspended in 20 µL supplemented P3 buffer (Lonza, V4XP-3032), mixed with RNPs, electroporated (pulse EH111), and rested in cRPMI for 15 min at 37°C. In parallel, lentiviral particles were loaded onto Retronectin-coated 24-wells (Takara, T100A) by centrifugation of concentrated lentiviral supernatants (2h, 2,000 g, 32°C). Rested CD8$^+$ T cells were added to lentivirus-coated wells and spinoculated (30 min, 800 g, 32°C). Transduced CD8$^+$ T cells were cultured in cRPMI containing IL-7 and IL-15 for 4 to 10 days before evaluation of transduction efficiency and tumor reactivity screening.

Flow cytometry. Cells were stained with Zombie NIR viability dye (BioLegend, 423106) in PBS for 15 min at 4°C, washed and resuspended in FACS buffer (0.5% BSA in PBS), blocked with human TruStain FcX for 10 min at 4°C, then stained with surface antibodies for 30 min at 4°C. For intracellular staining, fixation and permeabilization were performed using the eBioscience FoxP3/Transcription Factor Staining Buffer kit (Invitrogen, 00-5523-00) per manufacturer's protocol. The following antibodies were used for flow cytometry: SV500 anti-human CD3 (clone SK7, BioLegend 344870), SB550 anti-human CD8 (clone SK1, BioLegend 344760), APC anti-human TCRα/β (clone IP26, BioLegend 306718), BV421 anti-human TCRα/β (clone IP26, BioLegend 306722), AF700 anti-human CD69 (clone FN50, BioLegend 310922), APC anti-human CD137 (clone S18014C, BioLegend 300806), APC anti-human SOX10 (clone SP267, Abcam ab310845), PE anti-human Melan-A (clone A103, Santa Cruz Biotechnology sc-20032), and BV711 anti-mouse TCRβ (clone H57-597, BioLegend 109243). Samples were acquired on a Cytek Aurora. FlowJo v10.10.0 was used for data analysis and visualization.

Tumor reactivity screening of TCR-engineered T cells. Primary melanoma cells from patient 2 were thawed, expanded, and seeded into a flat-bottom 96-well plate (50,000 cells/well) in cRPMI. Melanoma cells were incubated for 4 h at 37°C and 5% CO_2 to allow for attachment, after which TCR-engineered CD8$^+$ T cells were added (250,000 cells/well) and co-cultured for 16 h. Wells without melanoma cells were included as negative controls. Following co-culture, upregulation of CD137 among TCR-engineered (human TCRα/β$^-$ and mouse TCRβ$^+$) CD8$^+$ T cells was measured by flow cytometry as an indicator of reactivity toward target tumor cells.

Study approval. This retrospective cohort study was conducted under Washington University School of Medicine Human Research Protections Office (HRPO) approved protocols in accordance with the principles outlined in the Declaration of Helsinki. Informed consent was obtained for clinical data analyses, genomic analyses, and clinical photography.

Data availability. Sequencing data is available at GEO (GSE350284)

AI disclosure. The authors declare that no AI tools were used in the original research or writing of this article

SUPPLEMENTAL TABLE

	T-VEC	T-VEC+Bini
**Sex**		
Female	4 (57.1%)	5 (71.4%)
Male	3 (42.9%)	2 (28.6%)
**Age at T-VEC initiation**		
Median (range)	58 (50-82)	64 (36-77)
**Tumor subtype**		
Cutaneous	6 (85.7%)	5 (71.4%)
Acral	1 (14.3%)	1 (14.3%)
Mucosal	0 (0%)	1 (14.3%)
**History of distant metastases**		
Yes	1 (14.3%)	5 (71.4%)
No	6 (85.7%)	2 (28.6%)
**Presence of non-injectable lesions at T-VEC start**		
Yes	1 (14.3%)	5 (71.4%)
No	6 (85.7%)	2 (28.6%)
**Brain metastases**		
Yes	0 (0%)	3 (42.9%)
No	7 (100%)	4 (57.1%)
**Prior lines of systemic therapy***		
0	3 (42.9%)	0 (0%)
1	3 (42.9%)	0 (0%)
2	1 (14.3%)	6 (85.7%)
3+	0 (0%)	1 (14.3%)
**Prior immune checkpoint blockade**		
Yes	4 (57.1%)	7 (100%)
No	3 (42.9%)	0 (0%)
**Concurrent systemic therapy***		
Nivolumab	1 (14.3%)	0 (0%)
Pembrolizumab	2 (28.6%)	0 (0%)
Relatlimab/nivolumab	1 (14.3%)	0 (0%)
Binimetinib	0 (0%)	6 (85.7%)
Nivolumab + binimetinib	0 (0%)	1 (14.3%)
None	3 (42.9%)	0 (0%)
**Clinical response***		
Complete (CR)	2 (28.6%)	6 (85.7%)
Partial (PR)	2 (28.6%)	0 (0%)
Stable disease (SD)	0 (0%)	0 (0%)
Progressive disease (PD)	3 (42.9%)	1 (14.3%)

Table S1: Clinical characteristics and outcomes in patients treated with T-VEC alone or T-VEC plus binimetinib. *Some columns add up to >100% due to rounding

SUPPLEMENTAL FIGURES

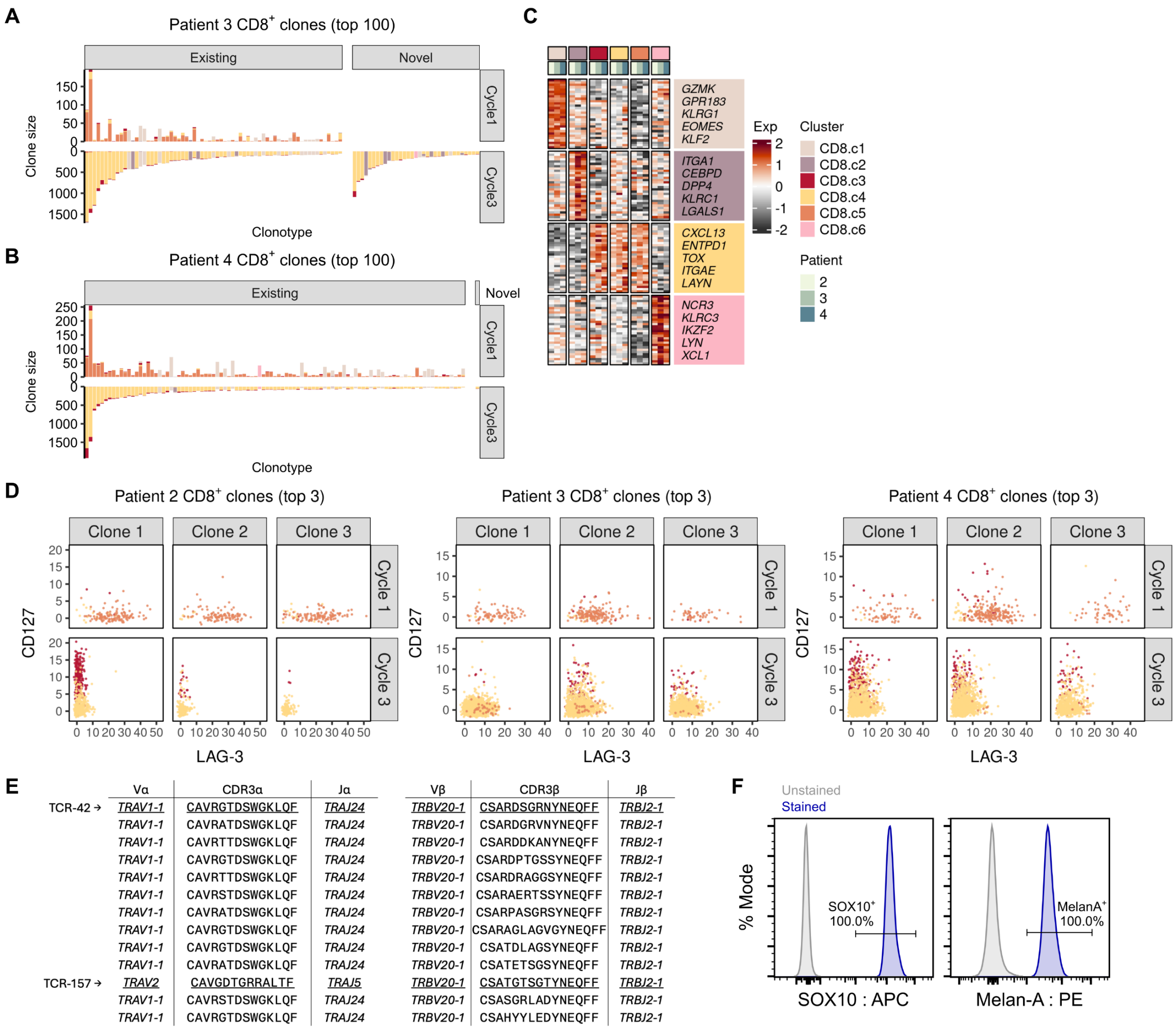

Figure S1. Pre-existing exhausted CD8+ T cell clones are found as cytotoxic effectors following combined T-VEC and binimetinib therapy. (**A-B**) Stacked bar plots depicting phenotypic distribution of expanded CD8+ T cell clones from Patient 3 (**A**) and Patient 4 (**B**). (**C**) Heatmap indicating differentially genes between c1 (effector memory), c2 (resident memory), c4 (cytotoxic), and c6 (NK-like) populations. (**D**) Shifts in surface phenotype across timepoints in selected representative CD8+ T cell clones. (**E**) Group of highly similar TCR sequences among Patient 2 CD8+ T cell clones. (**F**) Histograms displaying flow cytometric validation of melanoma marker expression in primary tumor cell line derived from the pre-treatment specimen of Patient 2.

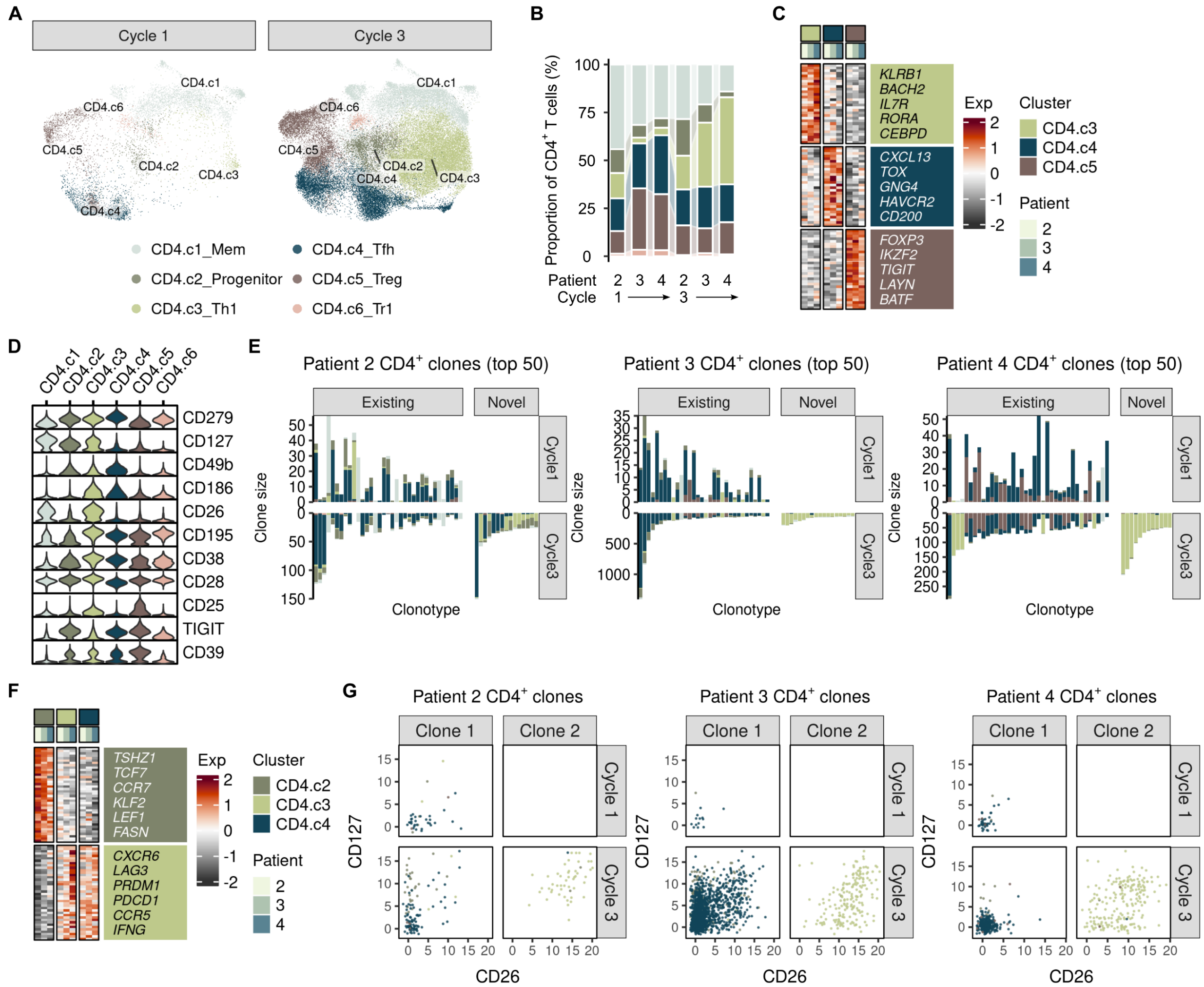

Figure S2. CD4⁺ T cell clonal dynamics following combined T-VEC and binimetinib therapy. (**A**) UMAPs of pre- and on-treatment intratumoral CD4⁺ T cells from patients 2-4. Mem/Tfh/Tr1, memory/follicular helper/type 1 regulatory T cell. (**B**) Stacked bar plots depicting sample-wise phenotypic distribution of CD4⁺ T cells. (**C**) Heatmap of differentially expressed genes between Th1, Tfh, and Tr1 subsets. (**D**) Violin plots of surface protein expression among CD4⁺ T cell subsets. (**E**) Stacked bar plots depicting phenotypic distribution of expanded CD4⁺ T cell clones. (**F**) Heatmap of differentially expressed genes between progenitor and differentiated CD4⁺ T cell states. (**G**) Surface phenotype across timepoints of selected Tfh- and Th1-biased CD4⁺ T cell clones.

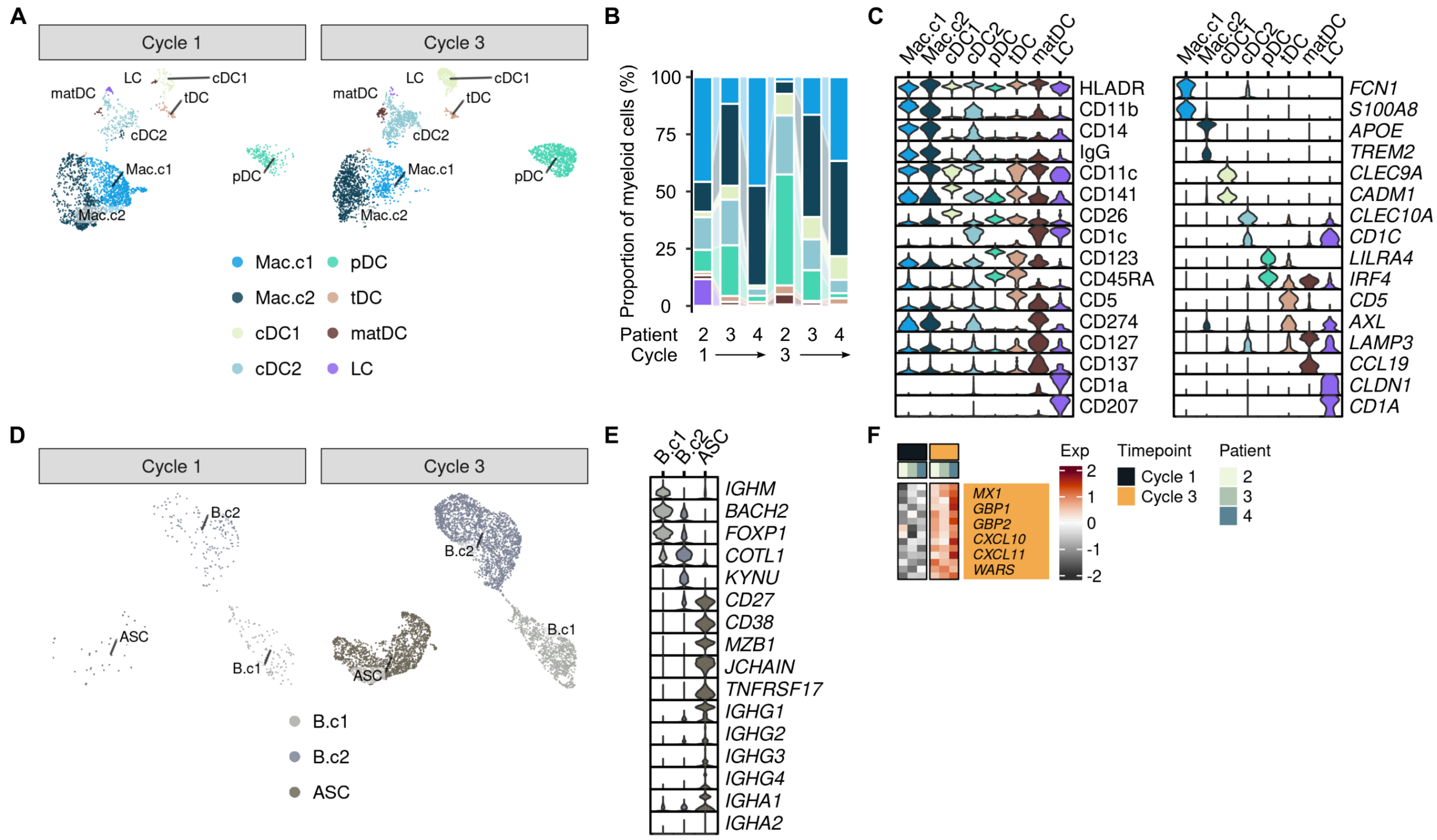

Figure S3. Changes in myeloid, B/plasma, and endothelial cell compartments following combined T-VEC and binimetinib therapy. (**A**) UMAPs of pre- and on-treatment intratumoral myeloid cells. Mac/cDC/pDC/tDC/matDC/LC, macrophage/conventional DC/plasmacytoid DC/transitional DC/mature DC/Langerhans cell from patients 2-4. (**B**) Stacked bar plots depicting sample-wise phenotypic distribution of myeloid cells. (**C**) Violin plots of surface protein and gene expression among myeloid cell subsets. (**D**) UMAPs of pre- and on-treatment intratumoral B and antibody-secreting cells (ASCs). (**E**) Violin plots of gene expression among B cells and ASCs. (**F**) Heatmap of differentially expressed genes between timepoints among blood endothelial cells.

SUPPLEMENTAL REFERENCES

1. Simon SCS, Müller V, Utikal JS. Case report: Therapeutic potential of T-VEC in combination with MEK inhibitors in melanoma patients with NRAS mutation. *Front Oncol*. 2023;13:1111119.

2. Zheng GXY, et al. Massively parallel digital transcriptional profiling of single cells. *Nat Commun*. 2017;8(1):14049.

3. Hao Y, et al. Dictionary learning for integrative, multimodal and scalable single-cell analysis. *Nat Biotechnol*. 2024;42(2):293–304.

4. McGinnis CS, et al. MULTI-seq: sample multiplexing for single-cell RNA sequencing using lipid-tagged indices. *Nat Methods*. 2019;16(7):619–626.

5. Mulè MP, Martins AJ, Tsang JS. Normalizing and denoising protein expression data from droplet-based single cell profiling. *Nat Commun*. 2022;13(1):2099.

6. Gu Z. Complex heatmap visualization. *iMeta*. 2022;1(3):e43.

7. Kuball J, et al. Facilitating matched pairing and expression of TCR chains introduced into human T cells. *Blood*. 2007;109(6):2331–2338.

8. Seki A, Rutz S. Optimized RNP transfection for highly efficient CRISPR/Cas9-mediated gene knockout in primary T cells. *J Exp Med*. 2018;215(3):985–997.
